# Implementation of the measure of case discussion complexity to guide selection of prostate cancer patients for multidisciplinary team meetings

**DOI:** 10.1002/cam4.6189

**Published:** 2023-05-31

**Authors:** Jessica Wihl, Victor Falini, Sixten Borg, Olof Stahl, Thomas Jiborn, Bjorn Ohlsson, Mef Nilbert

**Affiliations:** ^1^ Department of Clinical Sciences, Division of Oncology and Pathology Lund University Lund Sweden; ^2^ Regional Cancer Centre South, Region Skåne Lund Sweden; ^3^ Department of Hematology, Oncology and Radiation Physics Skåne University Hospital Lund Sweden; ^4^ Health Economics Unit, Department of Clinical Sciences in Malmö Lund University Lund Sweden; ^5^ Department of Urology Skåne University Hospital Malmö Sweden

**Keywords:** comorbidity, decision aid, multidisciplinary team, tumor board, prostate cancer

## Abstract

**Background:**

Multidisciplinary team meetings (MDTMs) provide an integrated team approach to ensure individualized and evidence‐based treatment recommendations and best expert advice in cancer care. A growing number of patients and more complex treatment options challenge MDTM resources and evoke needs for case prioritization. In this process, decision aids could provide streamlining and standardize evaluation of case complexity. We applied the recently developed Measure of Case Discussion Complexity, MeDiC, instrument with the aim to validate its performance in another healthcare setting and diagnostic area as a means to provide cases for full MDTM discussions.

**Methods:**

The 26‐item MeDiC instrument evaluates case complexity and was applied to 364 men with newly diagnosed prostate cancer in Sweden. MeDiC scores were generated from individual‐level health data and were correlated with clinicopathological parameters, healthcare setting, and the observed clinical case selection for MDTMs.

**Results:**

Application of the MeDiC instrument was feasible with rapid scoring based on available clinical data. Patients with high‐risk prostate cancers had significantly higher MeDiC scores than patients with low or intermediate‐risk cancers. In the total study, population affected lymph nodes and metastatic disease significantly influenced MDTM referral, whereas comorbidities and age did not predict MDTM referral. When individual patient MeDiC scores were compared to the clinical MDTM case selection, advanced stage, T3/T4 tumors, involved lymph nodes, presence of metastases and significant physical comorbidity were identified as key MDTM predictive factors.

**Conclusions:**

Application of the MeDiC instrument in prostate cancer may be used to streamline case selection for MDTMs in cancer care and may complement clinical case selection.

## Graphical abstract

Discussions of selection of patients that benefit the most of multidisciplinary team meetings (MDTM) are raised worldwide. Structures and validated instruments to select cases benefitting the most of discussions at MDTMs have been lacking until recently. The Measure of case Discussion Complexity instrument can be recommended as a clinical tool supporting a standardized selection of patients to multidisciplinary team meeting in an equal care pathway, used as a complement to clinical selection of prostate cancer patients.

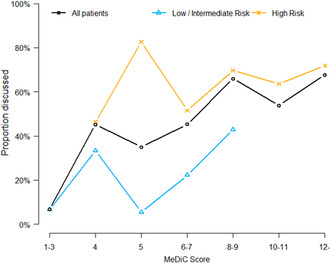

## 1 INTRODUCTION

In cancer care, the multidisciplinary team (MDT) approach has developed into standard of care with the aim to formulate an individualized, evidence‐based and relevant treatment recommendation. MDT‐based decision‐making increase likelihood for treatment according to guidelines and provide best expert opinion based on interdisciplinary and multiprofessional case evaluations and discussions. Multiple studies support an impact from MDT‐based case discussions on treatment plans and patient management, though the outcome differs between diagnoses and is influenced by tumor‐related as well as patient‐related factors such as stage, tumor biology, comorbidity, diagnostic complexity, and treatment options.^1^, ^2^

Prostate cancer is the leading male cancer type in western countries with more than 1.4 million diagnoses annually and over 700.000 deaths from the disease in Europe.^3^ Individualized therapies and refined treatment options contribute to increasing survival with 5‐year survival rates above 80%. Prostate cancer is a heterogenous disease with a multitude of different treatment options and a long and variable patient trajectory. This calls for multidisciplinary and comprehensive treatment recommendations and readiness to re‐evaluate and modify recommendations relative to clinical status and patient preferences. Management of prostate cancer by MDT is endorsed and supported by clinical guidelines and the European Cancer Organization, ECCO, defines a core MDT as a prerequisite for high‐quality prostate cancer care.^4^, ^5^ In prostate cancer, case discussions at MDTMs have been suggested to alter the diagnostic workup and/or the planned therapeutic strategy in one‐quarter to half of the cases. In metastatic cases, MDTM discussions have been shown to enhance treatment according to guidelines and improve outcomes and in low‐risk patients clinical benefit may relate to refraining from unnecessary treatments with reports of increased active surveillance based on MDT recommendations.^6^, ^7^, ^8^, ^9^, ^10^, ^11^, ^12^, ^13^, ^14^, ^15^

The broad implementation of MDTs in various diagnostic areas, a growing number of team participants, increasing cancer incidences and new and complex treatment options, however challenge MDTM resources. Waiting times for MDT have been documented; MDTM fatigue among participants has been reported; and patient safety considerations are raised related to limited case discussion time slots.^16^, ^17^, ^18^, ^19^, ^20^, ^21^, ^22^ To mitigate these challenges, some MDTs have developed prioritization strategies, for example, listing or mini‐MDTMs for standard cases and referral guidelines with selection of complex cases for full MDTMs. In several diagnostic areas, including prostate cancer, variable MDTM referral rates and discrepancies between referral guidelines and clinical practice have been documented, which motivates initiatives to support and standardize case selection to MDTMs.^11^, ^23^

The Measure of case Discussion Complexity, MeDiC, instrument was recently developed by experts in the UK to as a tool to select cases for MDTMs based on prescreen for case complexity factors. The MeDiC instrument was developed based on case complexity factors defined in breast cancer, colorectal cancer, and gynecological cancer.^24^ Evaluation of such instruments is relevant to perform in other contexts, for example, diagnostic areas and healthcare settings. This is important to explore the wider usability, acceptance and to identify needs for adjustments related to availability of information, principles for cases selection and relevance for the diagnostic and treatment‐determining multidisciplinary process. We aimed to assess applicability and external validity of the MeDiC instrument in the Swedish healthcare system and another diagnostic area, that is, patients with newly diagnosed prostate cancer. We also report on MeDiC scores in relation to clinicopathologic factors. We further aimed to investigate how MDTM case selection based on physician's judgment compared to application of the standardized MeDiC instrument.

### 1.1 Objective

The study aims to assess the performance of the MeDiC instrument for case selection to MDTM for patients with newly diagnosed prostate cancer, explore possible cut‐off scores, and compare outcomes between MeDiC‐based case selection and clinical case selection based on physicians' choice.

## 2 MATERIALS AND METHODS

### 2.1 Study design

The study has a retrospective, cross‐sectional design and is based on individual‐level clinical data. We aimed to study applicability, the external validity, and performance of MeDiC in a population‐based series of patients with newly diagnosed prostate cancer with comparison to clinically implemented case selection for full MDTM discussions.

### 2.2 Context and patients

In Sweden, diagnosis‐specific standardized care pathways have been introduced for most cancer types to grant efficient, evidence‐based and timely diagnostics. Patients with suspected prostate cancer, as defined by specified symptoms and/or digital rectal examination with findings suspicious for malignancy and/or increased PSA values, can be referred for diagnostic work‐up and treatment recommendations through the standardized care pathway. Low/intermediate‐risk tumor is confined to the prostate (T1c‐T2c), the PSA is below 20, and/or Gleason score 6–7. High‐risk tumors extend outside the prostate (T3/T4), the PSA >20, or/and Gleason 8–10. Recurrencies and metastatic prostate cancer are not managed through the standardized care pathways and were thus not included in our study.

In Sweden, national treatment guidelines recommend case discussion at MDTM for all patients with relevant comorbidities and for all high‐risk or metastatic prostate cancers with the additional requirement that patients should have an expected survival of five years. Region Skåne, with a population of 1.4 million, has one University hospital and four regional hospitals that diagnose and treat prostate cancer. All hospitals refer patients to the same MDTM that is held weekly with physical attendance at the University hospital and video‐based participation from regional hospitals. In addition, the University Hospital offers a joint urology and oncology outpatient clinic aimed to provide joint evaluations of newly diagnosed patients, complex cases and to inform and include patients in clinical trials, but patients referred to the joint clinic were not considered in the current study.

During 2020, 1014 men were diagnosed with prostate cancer in the southern Swedish Region Skåne. Of these, 548 were diagnosed through the standardized care pathway.^25^ In order to collect data from different healthcare settings, we selected all 221 patients diagnosed at regional hospitals and 143 randomly selected patients diagnosed at the University hospital. Clinical records were reviewed during the period July to October 2021 by an expert monitor (J.C.) to collect relevant clinical data as part of validation work on the prostate cancer care pathway.

### 2.3 Instrument and data collection

**FIGURE 1:**
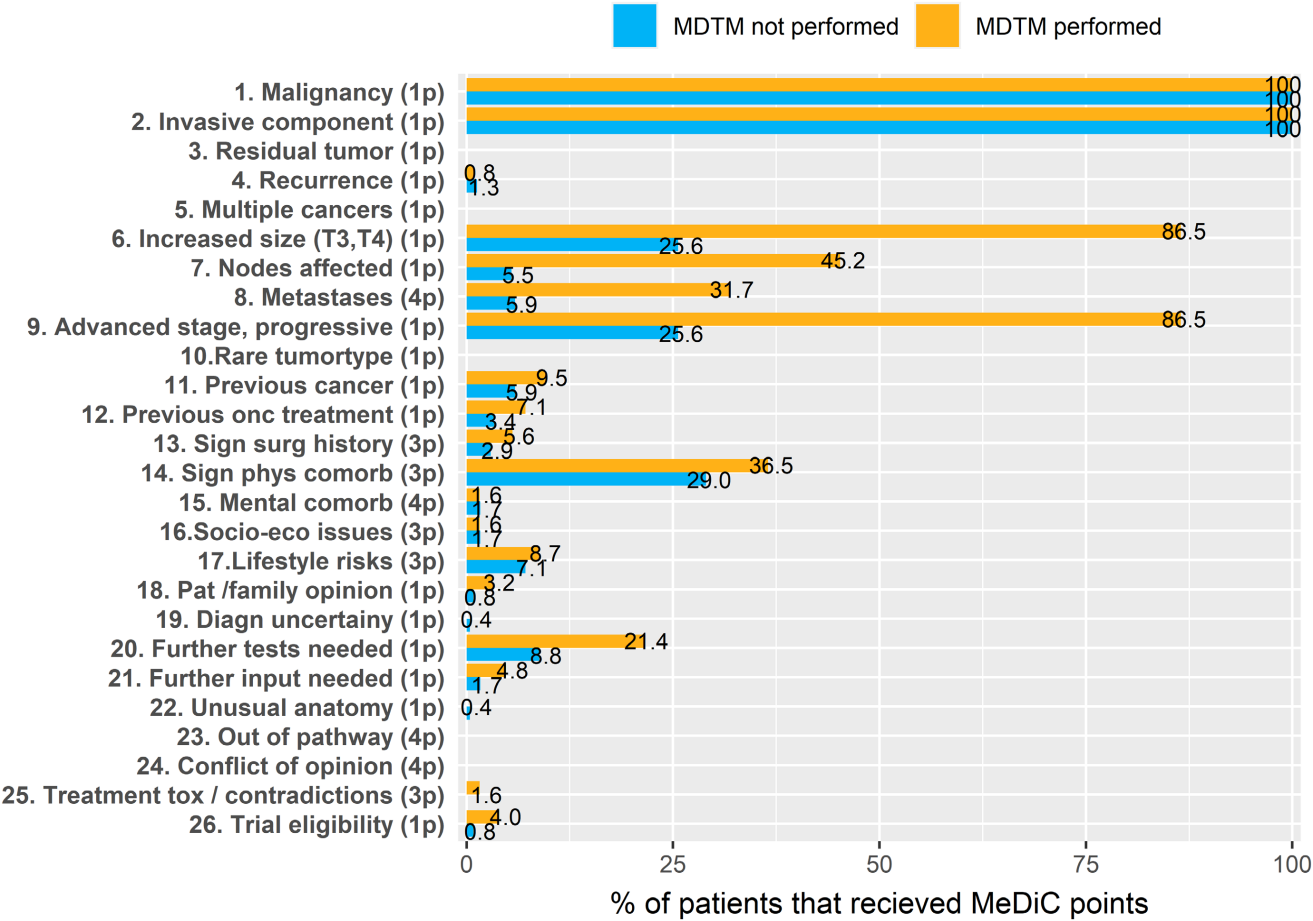
Percentage distribution of scores for the various MeDiC parameters in relation to patients that were selected for MDT‐based case discussions (orange) or not (blue). The items have different weight from 1 (lowest) to 4 (highest), in relation to their scope of case complexity, for example, metastatic disease 4 points, significant comorbidity 3 points, conflict of opinion in MDT 3 points .The scoring of items 2, 6, and 9 are matched with prostate cancer risk groups.

The MeDiC instrument was developed in the UK as a means to select patients to MDTM discussions. Cases of breast cancer, colorectal cancer, and gynecological cancer were used for development of the instrument. MeDiC contains 26 items in the 3 areas: pathology, patient characteristics, and treatment factors.^24^ The items have different weight from 1 (lowest) to 4 (highest), in relation to their scope of case complexity, for example, metastatic disease 4 points, significant comorbidity 3 points, conflict of opinion in MDT 3 points (Figure 1). Since only newly diagnosed cases were considered, none of our patients obtained scores related to relapse (items 3, 4, and 25) (Figure 1).

Individual‐level health data were collected, including data on comorbidity, psychosocial factors, PSA value, Gleason score, T stage, lymph node involvement, and presence of metastases.

### 2.4 Data analysis/statistics

**FIGURE 2:**
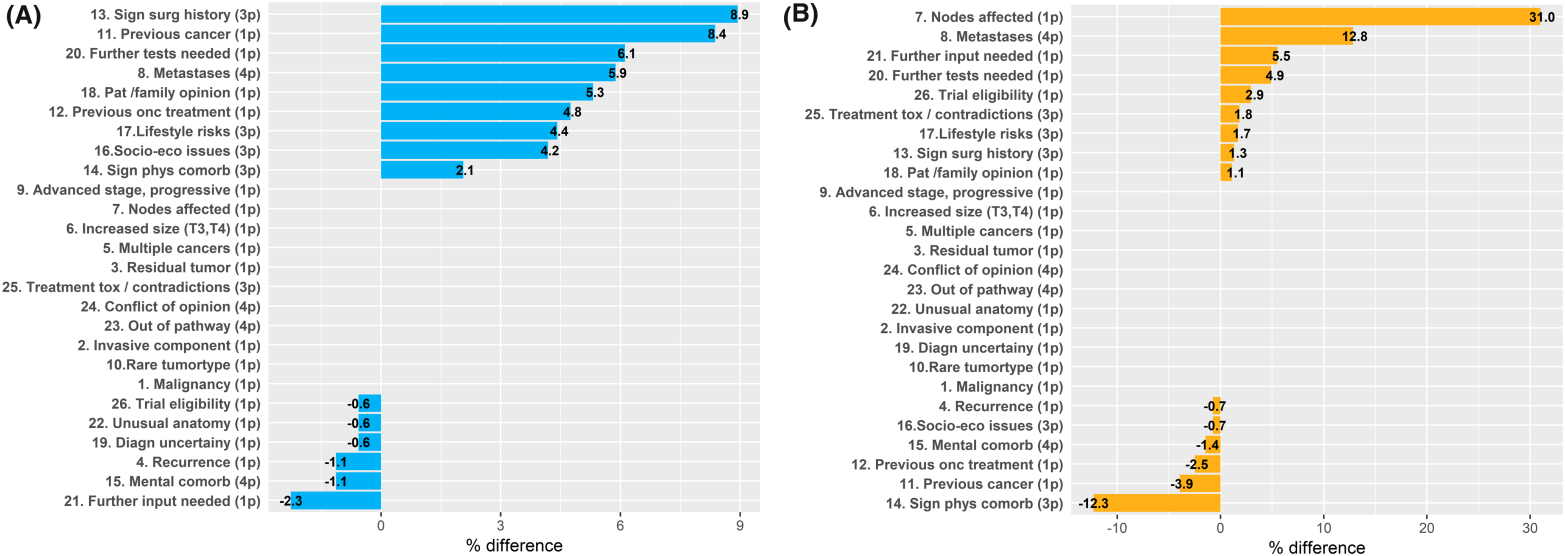
Importance of MeDiC factors for the clinical selection of patients for MDTM discussions. High‐risk tumors in blue to the left and low/intermediate‐risk tumors in yellow to the right.

**FIGURE 3:**
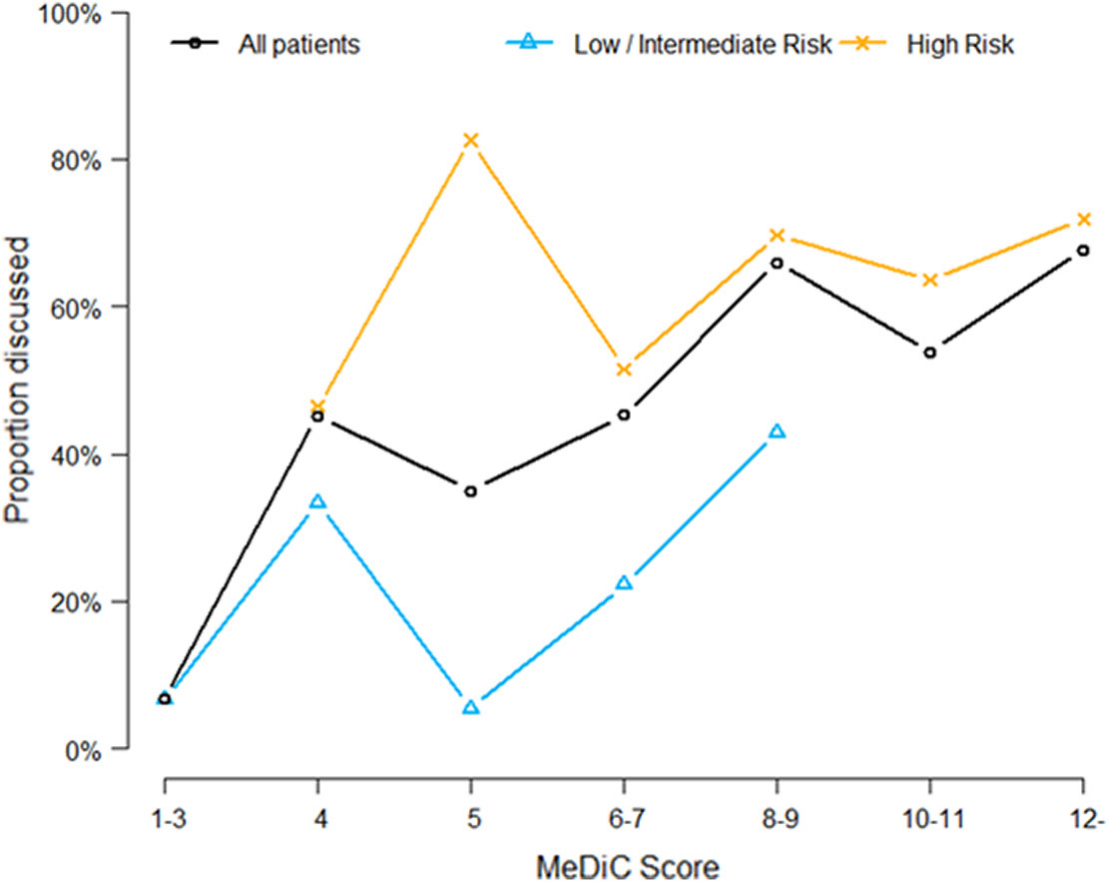
Proportion of patients with different MeDiC score discussed in Multidisciplinary Tumor Meetings, all patients (black graph), low/intermediate risk tumors (blue graph), and high‐risk tumors (orange graph). MeDiC scores were grouped in intervals to ensure at least three patients in each data point. Please note that some points in the diagram represent intervals of MeDiC score, for example “12‐” represent any score ≥ 12. Only few high scores were observed in the low/Intermediate‐risk group.

The items comprising the MeDiC instrument were collected from clinical record data, and a total MeDiC score was computed.^24^ Descriptive statistics of the study sample used Student's *t*‐test with significance level set at 0.05. The cases were classified high‐risk versus intermediate/low risk based on clinical data, ensuring that the scoring of item 2 (invasive component), 6 (increased T3/T4), and 9 (advanced stage) matched risk group classification. The influence of the various MeDiC items was plotted in falling order (Figure 2A,B). The MeDiC scores were further correlated to clinical case selection for MDTM discussion based on the difference in proportion scoring between the discussed and the non‐discussed groups (Figure 3). All data were analyzed with R version 4.0.5.^26^

### 2.5 Ethical approval

All data were handled anonymously and are presented at group level. The study was ethically reviewed and approved by the Lund University ethics committee, case no. 2019–04254/2021–01031.

## 3 RESULTS

In total, 364 patients were eligible for the study. Table S1 provides an overview of the study cohort. The median age at diagnosis was 72 years (range 46–94 years), 170 (47%) patients had high‐risk prostate cancers, 101 (27.7%) had T3/T4 tumors, and 54 (14.8%) had metastases at diagnosis. Comorbidities of importance for treatment recommendations were reported in 115 (32%) patients.

Application of the MeDiC instrument was found to be easy and feasible with rapid scoring based on available clinical data. The instrument highlights complexity factors relevant in a clinical handling of the patients. The population‐based cohort revealed a wide range of MeDiC scores with significantly higher scores in the high‐risk group compared to the low/intermediate‐risk group of prostate cancers (Figure S1).

In the total cohort, the MeDiC items most often scored were malignancy and invasive component, which were naturally present in all cases, followed by tumor size (46.7%), T3/T4 stage (46.7%), significant comorbidity (31.6%), affected lymph nodes (19.2%), presence of metastases (14.8%), and need for further investigations (13.2%) (Figure 1). The mean MeDiC score for the total study population was 5.48 (range 2–20) (Table S1). The mean MeDiC score for high‐risk patients discussed at a MDTM was 8.18 (range 4–20) compared to mean 7.59 (range 4–16) for high‐risk patients not discussed at a MDTM. The mean MeDiC score for low/intermediate‐risk patients discussed at a MDTM was 4.18 (range 2–8) compared to mean 3.20 (range 2–15) among low/intermediate‐risk patients not discussed at MDTM.

In total, 126 (34.6%) patients were referred to the regional MDTM based on physician's case selection. In addition, 27 (7.4%) of the patients diagnosed at the University hospital were evaluated at a joint urology and medical oncology interdisciplinary prostate cancer clinic. Of the 170 patients, 109 (64.1%) were selected for MDTM‐based case discussions though all high‐risk prostate cancer cases should, according to the national guidelines, be referred to a MDTM. In clinically based case selection for MDTMs, age (*p* < 0.001), affected lymph nodes (*p* < 0.001), and referring hospital (*p* = 0.002) significantly correlated with MDTM referral in high‐risk patients, whereas metastatic disease showed a significant correlation in low/intermediate risk patients (*p* = 000.1) (Table 1). Patient‐related information such as lifestyle risks, social economic issues or psychological issues and patient or family opinion were overall mentioned in 40 (11%) of the clinical files and did not correlate with MDTM referral. In the total study population affected lymph nodes and metastatic disease significantly influenced MDTM referral, whereas comorbidities and age did not predict referral (Table 1).

**TABLE 1:** Significant differences.

All patients				
	MDTM not performed (*N* = 238)	MDTM performed (*N* = 126)	Relative risk (95% CI)^a^	*p*‐value (χ^2^)^b^
Nodes affected	13 (5.5%)	57 (45.2%)	8.3 (4.72–14.53)	<0.001***
Metastatic disease	14 (5.9%)	40 (31.7%)	5.4 (3.05–9.53)	<0.001***
Comorbidity	69 (29.0%)	46 (36.5%)	1.26 (0.93–1.71)	0.142
Patient diagnosed at University Hospital	108 (45%)	35 (27.8%)	0.61 (0.45–0.84)	0.0011**
Age				*p*‐value (*t*‐test)
Mean (SD)	71.0 (9.85)	72.2 (6.52)	Not applicable	0.167
Median [Min, Max]	70.0 [46.0, 94.0]	74.0 [52.0, 85.0]		

High‐risk patients				
	MDTM not performed (*N* = 61)	MDTM performed (*N* = 109)	Relative risk (95% C.I.)^a^	*p*‐value (χ^2^)^b^
Nodes affected	13 (21.3%)	57 (52.3%)	2.45 (1.47–4.1)	<0.001***
Metastatic disease	14 (23.0%)	39 (35.8%)	1.56 (0.92–2.63)	0.083
Comorbidity	31 (50.8%)	42 (38.5%)	0.76 (0.54–1.07)	0.121
Patient diagnosed at University Hospital	28 (46%)	25 (23%)	0.5 (0.32–0.77)	0.002**
Age				*p*‐value (*t*‐test)
Mean (SD)	80.1 (8.70)	72.6 (6.53)	Not applicable	<0.001***
Median [Min, Max]	82.0 [57.0, 94.0]	74.0 [52.0, 85.0]		

Low/Intermediate‐risk patients				
	MDTM not performed (*N* = 177)	MDTM performed (*N* = 17)	Relative risk (95% C.I.)^a^	*p*‐value (χ^2^)^b^
Nodes affected	0 (0%)	0 (0%)	Not applicable	Not applicable
Metastatic disease	0 (0%)	1 (5.9%)	Not applicable	0.0012**
Comorbidity	5 (2.8%)	2 (11.8%)	1.1 (0.44–2.7)	0.84
Patient diagnosed at University Hospital	80 (45%)	10 (58%)	1.3 (0.85–2)	0.28
Age				*p*‐value (*t*‐test)
Mean (SD)	67.9 (8.15)	70.0 (6.15)	Not applicable	0.209
Median [Min, Max]	68.0 [46.0, 91.0]	69.0 [60.0, 82.0]		

^a^ 95% Confidence Interval.

^b^ Chi‐square test.

MeDiC items were further analyzed in relation to prostate cancer risk group. In patients with high‐risk tumors, affected lymph nodes (item 7) and presence of distant metastases (item 8) were the two items that most frequently correlated with case discussion at MDTM (Figure 2). In this group, patients selected for MDTM discussion were 2.5 times more likely to have affected lymph nodes and 1.6 times more likely to have metastatic disease compared to patients that were not selected for MDTM. In patients with low and intermediate risk tumors, significant surgical history, previous cancer, need for further test, presence of metastases, and patient/family opinion were the MeDiC scoring items that predicted case discussions at MDTMs (Figure 2).

The MeDiC instrument does not predefine cutoff levels since this may depend on the diagnosis and the clinical setting. In prostate cancer, high‐risk tumors do by definition obtain MeDiC scores ≥4. We explored the external validity between MeDiC scores and clinical selection for case discussion at MDTM in different risk groups and observed good overall correlation (Figure 3). The MeDiC score appears to offer an explanation to the selection of cases for MDTM, suggesting a threshold for MDTM around 8. High‐risk patients are often selected for MDTM regardless of MeDiC score, whereas low‐ and intermediate‐risk patients are selected in cases with higher MeDiC scores.

## 4 DISCUSSION

Requests for MDT case discussions are rapidly increasing in uro‐oncology motivated by growing patient volumes and expanding treatment options. The initial focus at MDTMs for prostate cancer related to radical treatment versus active surveillance, but with expanding treatment options and requests for tailored treatment plans recurrent case discussions are requested, for example, in complex and advanced cases, related to altered treatment intention, possibility for salvage treatment and choice of medical treatment.^5^, ^12^, ^27^, ^28^, ^29^ Resource constraints lead to quests for selection and prioritization of prostate cancers to MDTMs. International guidelines recommend MDT‐based case discussions for all prostate cancer patients, whereas national Swedish guidelines recommend discussion of patients with high‐risk tumors and minimum 5‐year expected survival. In our population‐based sample of newly diagnosed prostate cancers, 34.6%, including 64.1% of high‐risk tumors and 8.8% of low/intermediate‐risk tumors, obtained a recommendation from an MDTM, which demonstrates prioritization in clinical practice to handle workload and ensure timely treatment.^30^, ^31^

### 4.1 Case complexity factors

Several case complexity factors and the influence from comorbidity are consistent across cancer diagnoses, which should to some degree allow standardized evaluation of case evaluation and prioritization. The MeDiC instrument applies various factors to identify case complexity, but there is at present no consensus on how factors such as comorbidity, performance status, rare tumors morphology or molecular profile, borderline resectability, should be weighted.^21^, ^28^, ^32^ Our study is to our knowledge the first to apply and externally validate the MeDiC instrument outside of the UK, in prostate cancer and in patients diagnosed through the streamlined standardized care pathway.^24^ Application and evaluation of a newly developed instruments such as MeDiC in another healthcare system and diagnostic area are relevant to assess broader acceptance and benefit. Independent external implementation may also identify new aspect such as the performance in low‐risk versus high‐risk tumors here demonstrated. We aimed to assess feasibility of MeDiC application and assess case selection based on MeDiC scores versus physician's choice based on clinical parameters. In our material, a wide range of MeDiC scores were found with extensive inter‐patient variability and different profiles in to high‐risk versus low/intermediate‐risk tumors (Figure 1). The items that correlated with clinical selection for case discussion at MDTM were affected lymph nodes and distant metastases in the high‐risk group and surgical history, previous cancer, need for further test, metastases, and patient/family opinion in low/intermediate‐risk patients (Figure 2).

Comorbidity influences case selection for MDTM and treatment recommendations from the MDTM. The MeDiC instrument handles comorbidities as one item, and it does not distinguish between more general risks related to comorbidity and treatment‐specific issues related to surgical risk or to tolerance for medical treatments. We documented comorbidities in 29% of the patients that were not discussed at a MDTM and in 36.5% of the patients discussed at a MDTM, which suggest that comorbidities do not seem to be a major determinant of MDTM case selection (supplementary Table S1).

Elderly patients (>80 years) with high‐risk tumors were less often referred to a MDTMs, which may be explained by Swedish recommendations of considering remaining lifetime and treatment intent, but also a question of treatment modality. Surgery is, for example, seldom an option in elderly patients due to adverse complications of incontinence. Treatment decisions are then preferably made in the patient meeting rather than at MDTM. A lower likelihood for discussion of elderly patients with an adverse prognosis has been documented also in other MDT settings and selection of cases for MDTMs tend to favor patients eligible for multidisciplinary treatments but can also raise questions related to equity in care.^29^, ^33^, ^34^ Information about relevant comorbidities and performance status are important to take in account and to support decision‐making in elderly patients geriatric assessment tools may be relevant as a complement to clinical judgment.^35^

In Sweden, video‐based regional MDTMs have been established to provide easy and equitable access to interdisciplinary and multiprofessional case discussions also for patients managed at regional hospitals. It was therefore somewhat unexpected to find unbalanced geographical referral rates with significantly lower (27.8% versus 64.1%) MDTM referral rates for patients managed at the University hospital compared to regional hospitals. Equity in care should grant expert advice irrespective of healthcare setting, but disparities in prostate cancer care have been documented also in other healthcare systems and clinical settings.^8^, ^28^, ^36^ The lower MDTM referral at the University hospital could potentially be explained by patient management through the joint prostate cancer clinic served by urologists and medical oncologists, but since only 7.4% of the patients were referred to the joint clinic this is not a major explanation to the difference observed. Such clinics have, however, been reported to enhance shared decision‐making and positively influence outcomes, particularly in high‐risk, locally advanced disease based on data from the United States, France, and in Switzerland.^12^, ^37^, ^38^, ^39^, ^40^, ^41^

### 4.2 Strengths and limitations

To the best of our knowledge, this is the first study using the MeDiC instrument outside of the UK and in the prostate cancer context. The study included patients diagnosed through the standardized care pathway, managed in different healthcare settings and referred to the same virtual, regional MDTM. Retrospective review of patient files was conducted by one experienced, dedicated reviewer to grant consistent data collection. Limitations include retrospective study design and data based on clinical files, which may imply missing data due to lack of data in clinical records, for example, related to comorbidities. Further, our study focused on newly diagnosed prostate cancers, and we did not evaluate recurrencies or cases discussed because of local recurrencies or development of metastases.

### 4.3 Clinical implications

We conclude that application of the MeDiC instrument is feasible in prostate cancer care and can effectively be used to support selection of patients for MDTMs. MeDiC scores show large inter‐patient variability and high‐risk and low/intermediate‐risk tumors show different MeDiC profiles with correlations to clinical case selection. The findings suggest that structured selection and standardized prioritization are possible but needs to be targeted to the specific diagnosis and healthcare setting. The results also reveal suboptimal adherence to MDTM referral principles and thus serves as a reminder to the impact from continuous evaluation and development of MDTM services to ensure equity of care and effective use of resources. Further studies of the MeDiC instrument in other healthcare settings and diagnostic areas and prospective data collection would be relevant for further development, optimization, and definition of relevant cutoff levels.

## 5 CONCLUSIONS

We conclude that assessment of case complexity using the MeDiC instrument is feasible. The instrument defined key parameters for patient referral to MDTM, which in patients with high‐risk tumors were affected lymph nodes and presence of distant metastases. MeDiC scores and clinical case selection for MDTM showed good correlation with a suggested threshold around 8. We suggest that MeDiC can be a valuable addition to complement clinical selection of prostate cancer patients for MDTM.

## AUTHOR CONTRIBUTIONS

**Jessica Wihl:** Conceptualization (lead); data curation (lead); formal analysis (lead); funding acquisition (supporting); investigation (lead); methodology (lead); project administration (lead); resources (supporting); software (supporting); supervision (supporting); validation (lead); visualization (supporting); writing – original draft (lead); writing – review and editing (lead). **Victor Falini:** Conceptualization (supporting); data curation (lead); formal analysis (lead); funding acquisition (supporting); investigation (supporting); methodology (supporting); project administration (supporting); resources (supporting); software (lead); supervision (supporting); validation (supporting); visualization (lead); writing – original draft (lead); writing – review and editing (supporting). **Sixten Borg:** Conceptualization (supporting); data curation (lead); formal analysis (lead); funding acquisition (supporting); investigation (supporting); methodology (lead); project administration (supporting); resources (supporting); software (lead); supervision (supporting); validation (lead); visualization (lead); writing – original draft (lead); writing – review and editing (lead). **Olof Ståhl:** Conceptualization (equal); data curation (supporting); formal analysis (equal); funding acquisition (supporting); investigation (supporting); methodology (equal); project administration (supporting); resources (supporting); software (supporting); supervision (equal); validation (lead); visualization (supporting); writing – original draft (equal); writing – review and editing (supporting). **Thomas Jiborn:** Conceptualization (equal); data curation (supporting); formal analysis (equal); funding acquisition (supporting); investigation (supporting); methodology (equal); project administration (supporting); resources (supporting); software (supporting); supervision (supporting); validation (supporting); visualization (equal); writing – original draft (supporting); writing – review and editing (supporting). **Bjorn Ohlsson:** Conceptualization (lead); data curation (equal); formal analysis (supporting); funding acquisition (lead); investigation (equal); methodology (equal); project administration (supporting); resources (equal); software (supporting); supervision (equal); validation (equal); visualization (supporting); writing – original draft (equal); writing – review and editing (supporting). **Mef Nilbert:** Conceptualization (lead); data curation (lead); formal analysis (lead); funding acquisition (lead); investigation (equal); methodology (lead); project administration (equal); resources (lead); software (equal); supervision (lead); validation (lead); visualization (equal); writing – original draft (lead); writing – review and editing (equal).

## FUNDING INFORMATION

This research was funded by the *Kamprad Cancer Fund* and the Regional Cancer Centre South, Region Skåne.

## CONFLICT OF INTEREST STATEMENT

The authors declare no conflict of interest.

## INSTITUTIONAL REVIEW BOARD STATEMENT

The study was conducted in accordance with the Declaration of Helsinki, ethically reviewed and approved by the Lund University ethics committee, case no. 2019–04254/2021–01031.

## INFORMED CONSENT STATEMENT

Patient consent was waived due to data were handled on a group level, deidentified, individual patients are not possible to identify.

## Supporting information


Figure S1.

Table S1.

## Data Availability

Raw data are available from the authors based on reasonable request and with respect for the GDPR rules for protection of personal integrity.

## References

[1] Lamb BW , Brown KF , Nagpal K , Vincent C , Green JS , Sevdalis N . Quality of care management decisions by multidisciplinary cancer teams: a systematic review. Ann Surg Oncol. 2011;18(8):2116‐2125. doi:10.1245/s10434-011-1675-6 21442345

[2] Soukup T , Lamb BW , Arora S , Darzi A , Sevdalis N , Green JS . Successful strategies in implementing a multidisciplinary team working in the care of patients with cancer: an overview and synthesis of the available literature. J Multidiscip Healthcare. 2018;11:49‐61. doi:10.2147/jmdh.s117945 PMC5783021 29403284

[3] Sung H , Ferlay J , Siegel RL , et al. Global cancer statistics 2020: GLOBOCAN estimates of incidence and mortality worldwide for 36 cancers in 185 countries. CA Cancer J Clin. 2021;71(3):209‐249. doi:10.3322/caac.21660 33538338

[4] Mottet N , van den Bergh RCN , Briers E , et al. EAU‐EANM‐ESTRO‐ESUR‐SIOG guidelines on prostate Cancer‐2020 update. Part 1: screening, diagnosis, and local treatment with curative intent. Eur Urol. 2021;79(2):243‐262. doi:10.1016/j.eururo.2020.09.042 33172724

[5] Brausi M , Hoskin P , Andritsch E , et al. ECCO essential requirements for quality cancer care: prostate cancer. Crit Rev Oncol Hematol. 2020;148:102861. doi:10.1016/j.critrevonc.2019.102861 32151466

[6] De Luca S , Fiori C , Tucci M , et al. Prostate cancer management at an Italian tertiary referral center: does multidisciplinary team meeting influence diagnostic and therapeutic decision‐making process? A snapshot of the everyday clinical practice. Minerva Urol Nefrol. 2019;71(6):576‐582. doi:10.23736/s0393-2249.19.03231-4 31487974

[7] Rao K , Manya K , Azad A , et al. Uro‐oncology multidisciplinary meetings at an Australian tertiary referral Centre‐impact on clinical decision‐making and implications for patient inclusion. BJU Int. 2014;114(Suppl 1):50‐54. doi:10.1111/bju.12764 25070295

[8] Kočo L , Weekenstroo HHA , Lambregts DMJ , et al. The effects of multidisciplinary team meetings on clinical practice for colorectal, lung, prostate and breast cancer: a systematic review. Cancers (Basel). 2021;13(16):4159. doi:10.3390/cancers13164159 34439312 PMC8394238

[9] El Khoury R , Chahrouri M , Hachem C , Abi Zeid J , El Alam P , Abdessater M . Evaluation of multidisciplinary team meetings IN URO‐oncology. J Med Liban. 2016;64(2):84‐90. 30452145

[10] Bayoud Y , Loock PY , Messaoudi R , et al. Prostate cancer: what about reproducibility of decision made at multidisciplinary team management? Urol J. 2015;12(2):2078‐2082. 25923152

[11] Brown B , Young J , Smith DP , et al. A multidisciplinary team‐oriented intervention to increase guideline recommended care for high‐risk prostate cancer: a stepped‐wedge cluster randomised implementation trial. Implement Sci. 2018;13(1):43. doi:10.1186/s13012-018-0733-x 29530071 PMC5848547

[12] Holmes A , Kelly BD , Perera M , Eapen RS , Bolton DM , Lawrentschuk N . A systematic scoping review of multidisciplinary cancer team and decision‐making in the management of men with advanced prostate cancer. World J Urol. 2021;39(2):297‐306. doi:10.1007/s00345-020-03265-1 32500304

[13] Aizer AA , Paly JJ , Zietman AL , et al. Multidisciplinary care and pursuit of active surveillance in low‐risk prostate cancer. J Clin Oncol. 2012;30(25):3071‐3076. doi:10.1200/jco.2012.42.8466 22851571

[14] Saad F , Canil C , Finelli A , et al. Controversial issues in the management of patients with advanced prostate cancer: results from a Canadian consensus forum. Can Urol Assoc J. 2020;14(4):E137‐E149. doi:10.5489/cuaj.6082 31702544 PMC7124178

[15] Zhu S , Chen J , Ni Y , et al. Dynamic multidisciplinary team discussions can improve the prognosis of metastatic castration‐resistant prostate cancer patients. Prostate. 2021;81(11):721‐727. doi:10.1002/pros.24167 34028061 PMC8362088

[16] Lamb BW , Sevdalis N , Benn J , Vincent C , Green JS . Multidisciplinary cancer team meeting structure and treatment decisions: a prospective correlational study. Ann Surg Oncol. 2013;20(3):715‐722. doi:10.1245/s10434-012-2691-x 23064794

[17] Ke KM , Blazeby JM , Strong S , Carroll FE , Ness AR , Hollingworth W . Are multidisciplinary teams in secondary care cost‐effective? A systematic review of the literature. Cost Eff Resour Alloc. 2013;11(1):7. doi:10.1186/1478-7547-11-7 23557141 PMC3623820

[18] Alexandersson N , Rosell L , Wihl J , Ohlsson B , Steen Carlsson K , Nilbert M . Determinants of variable resource use for multidisciplinary team meetings in cancer care. Acta Oncol. 2018;57(5):675‐680. doi:10.1080/0284186x.2017.1400682 29199517

[19] Munro AJ . Multidisciplinary team meetings in cancer care: an idea whose time has gone? Clin Oncol (R Coll Radiol). 2015;27(12):728‐731. doi:10.1016/j.clon.2015.08.008 26365047

[20] Reyes Veliz A , Gray J , Karnon J . Economics of multidisciplinary teams in oncology: a scoping review protocol. JBI Evid Synth. 2020;18(6):1285‐1291. doi:10.11124/jbisrir-d-19-00103 32813376

[21] Edney LCGJ , Karnon J . A scoping review of the economics of multidisciplinary teams in oncology care. J Cancer Policy. 2019;26:100257. doi:10.1016/j.jcpo.2020.100257

[22] Soukup T , Gandamihardja TAK , McInerney S , Green JSA , Sevdalis N . Do multidisciplinary cancer care teams suffer decision‐making fatigue: an observational, longitudinal team improvement study. BMJ Open. 2019;9(5):e027303. doi:10.1136/bmjopen-2018-027303 PMC6549703 31138582

[23] Lamb BW , Jalil RT , Sevdalis N , Vincent C , Green JS . Strategies to improve the efficiency and utility of multidisciplinary team meetings in urology cancer care: a survey study. BMC Health Serv Res. 2014;14:377. doi:10.1186/1472-6963-14-377 25196248 PMC4162937

[24] Soukup T , Morbi A , Lamb BW , et al. A measure of case complexity for streamlining workflow in multidisciplinary tumor boards: mixed methods development and early validation of the MeDiC tool. Cancer Med. 2020;9(14):5143‐5154. doi:10.1002/cam4.3026 32476281 PMC7367630

[25] Socialstyrelsen cancerregistret . Statistikdatabas för cancer. 2020. https://www.socialstyrelsen.se/statistik‐och‐data/register/cancerregistret/.

[26] R Core Team. R Core Team (2021). R: A Language and Environment for Statistical Computing. R Foundation for Statistical Computing. URL https://www.R‐project.org/.

[27] Aizer AA , Paly JJ , Efstathiou JA . Multidisciplinary care and management selection in prostate cancer. Semin Radiat Oncol. 2013;23(3):157‐164. doi:10.1016/j.semradonc.2013.01.001 23763881

[28] Warner R , Hoinville L , Pottle E , Taylor C , Green J . Refocusing cancer multidisciplinary team meetings in the United Kingdom: comparing urology with other specialties. Ann R Coll Surg Engl. 2021;103(1):10‐17. doi:10.1308/rcsann.2020.0212 32981347 PMC7705157

[29] Scarberry K , Ponsky L , Cherullo E , et al. Evaluating the impact of the genitourinary multidisciplinary tumour board: should every cancer patient be discussed as standard of care? Can Urol Assoc J. 2018;12(9):E403‐E408. doi:10.5489/cuaj.5150 29787374 PMC6143504

[30] Winters DA , Soukup T , Sevdalis N , Green JSA , Lamb BW . The cancer multidisciplinary team meeting: in need of change? History, challenges and future perspectives. BJU Int. 2021;128(3):271‐279. doi:10.1111/bju.15495 34028162

[31] Atwell D , Vignarajah DD , Chan BA , et al. Referral rates to multidisciplinary team meetings: is there disparity between tumour streams? J Med Imaging Radiat Oncol. 2019;63(3):378‐382. doi:10.1111/1754-9485.12851 30623607

[32] Acher PL , Young AJ , Etherington‐Foy R , McCahy PJ , Deane AM . Improving outcomes in urological cancers: the impact of "multidisciplinary team meetings". Int J Surg (London, England). 2005;3(2):121‐123. doi:10.1016/j.ijsu.2005.06.006 17462272

[33] Walraven JEW , Desar IME , van der Hoeven JJM , et al. Analysis of 105.000 patients with cancer: have they been discussed in oncologic multidisciplinary team meetings? A nationwide population‐based study in The Netherlands. Eur J Cancer. 2019;121:85‐93. doi:10.1016/j.ejca.2019.08.007 31563730

[34] Rollet Q , Bouvier V , Moutel G , et al. Multidisciplinary team meetings: are all patients presented and does it impact quality of care and survival–a registry‐based study. BMC Health Serv Res. 2021;21(1):1032. doi:10.1186/s12913-021-07022-x 34592971 PMC8485542

[35] Zereshkian A , Cao X , Puts M , et al. Do Canadian radiation oncologists consider geriatric assessment in the decision‐making process for treatment of patients 80 years and older with non‐metastatic prostate cancer? ‐ National Survey. J Geriatr Oncol. 2019;10(4):659‐665. doi:10.1016/j.jgo.2019.01.015 30952518

[36] Hoinville L , Taylor C , Zasada M , Warner R , Pottle E , Green J . Improving the effectiveness of cancer multidisciplinary team meetings: analysis of a national survey of MDT members' opinions about streamlining patient discussions. BMJ Open Quality. 2019;8(2):e000631. doi:10.1136/bmjoq-2019-000631 PMC6567952 31259288

[37] Betschart P , Babst C , Schmid S , et al. Shared decision‐making for patients with advanced urological malignancies: evaluation of a joint urological‐oncological clinic model. Oncol Res Treat. 2019;42(7–8):366‐374. doi:10.1159/000499721 31170721

[38] Patrikidou A , Maroun P , Patard JJ , et al. Helping patients make informed decisions. Two‐year evaluation of the Gustave Roussy prostate cancer multidisciplinary clinic. Clin Transl Radiat Oncol. 2018;12:28‐33. doi:10.1016/j.ctro.2018.07.001 30094353 PMC6072649

[39] Gomella LG , Lin J , Hoffman‐Censits J , et al. Enhancing prostate cancer care through the multidisciplinary clinic approach: a 15‐year experience. J Oncol Pract. 2010;6(6):e5‐e10. doi:10.1200/jop.2010.000071 21358951 PMC2988679

[40] Borras JM , Albreht T , Audisio R , et al. Policy statement on multidisciplinary cancer care. Eur J Cancer. 2014;50(3):475‐480. doi:10.1016/j.ejca.2013.11.012 24321260

[41] Nazim SM , Fawzy M , Bach C , Ather MH . Multi‐disciplinary and shared decision‐making approach in the management of organ‐confined prostate cancer. Arab J Urol. 2018;16(4):367‐377. doi:10.1016/j.aju.2018.06.008 30534434 PMC6277278

