# Supplementary figures and images for: Implementation of the measure of case discussion complexity to guide selection of prostate cancer patients for multidisciplinary team meetings
Source: Cancer Med. 2023 May 31;12(14):15149–58. doi: 10.1002/cam4.6189 (PMC10417062; doi:10.1002/cam4.6189)

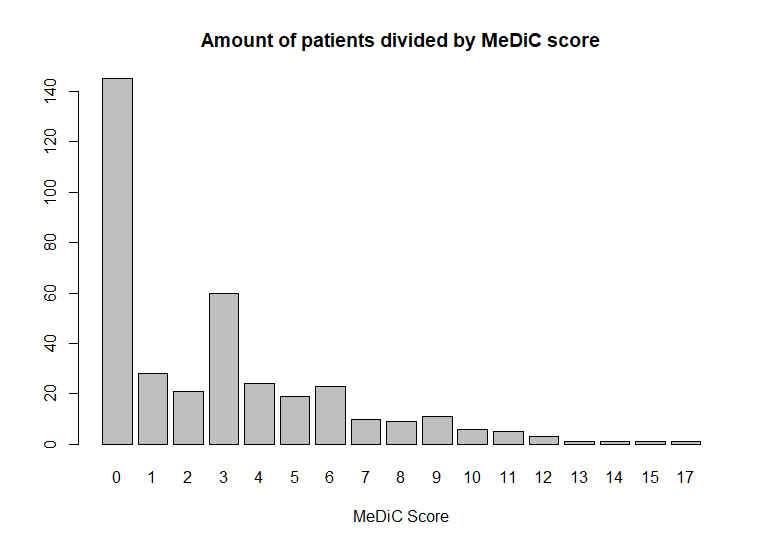

Supplement: Supplementary file 1 — Figure S1. [file CAM4-12-15149-s002.zip › CAM4_6189_Supplementary figure 1a.png]

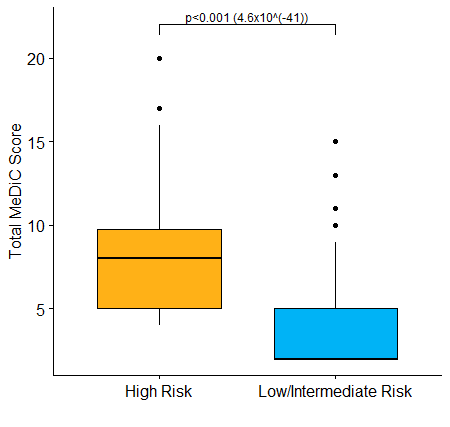

Supplement: Supplementary file 1 — Figure S1. [file CAM4-12-15149-s002.zip › CAM4_6189_supplementary figure 1b.png]
